# Immunomodulatory effect of an isolated fraction from *Tinospora crispa* on intracellular expression of INF-γ, IL-6 and IL-8

**DOI:** 10.1186/1472-6882-14-205

**Published:** 2014-06-27

**Authors:** Walaa Najm Abood, Iman Fahmi, Mahmood Ameen Abdulla, Salmah Ismail

**Affiliations:** 1 Department of Biomedical Science, Faculty of Medicine, University of Malaya, 50603 Kuala Lumpur, Malaysia; 2 Department of Microbiology and Immunology, Faculty of Medicine, University of Diyala, Baqubah, Iraq; 3 Department of Molecular medicine, Faculty of Medicine, University of Malaya, 50603 Kuala Lumpur, Malaysia; 4 Institute of Biological Science, Faculty of Science, University of Malaya, 50603 Kuala Lumpur, Malaysia

**Keywords:** IL-6, IL-8, Immunomodulatory, INF-γ, *Tinospora crispa*

## Abstract

**Background:**

Immunomodulators are substances that modify immune system response to a threat. Immunomodulators modulate and potentiate the immune system, keeping it highly prepared for any threat. The immunomodulatory effect of the traditional medicine *Tinospora crispa* is investigated in this work.

**Methods:**

*T. crispa* ethanol extract was fractionated by using different solvents. The ethanol extract and effective isolated fraction were used to investigate the potential immunomodulatory effect of different *T. crispa* doses ranging from 25 μg/mL to 1000 μg/mL on RAW 246.7 cells by detecting intracellular INF-γ, IL-6, and IL-8 expressions. The antioxidant activity of *T. crispa* was evaluated through FRAP and DPPH. The total phenolic and total flavonoid contents were also quantified.

**Results:**

Results show that *T. crispa* extract has higher antioxidant potential than ascorbic acid. The FRAP value of *T. crispa* extract is 11011.11 ± 1145.42 μmol Fe^+2^/g, and its DPPH inhibition percentage is 55.79 ± 7.9, with 22 μg/mL IC50. The results also reveal that the total phenolic content of *T. crispa* extract is 213.16- ± 1.31 mg GAE/g dry stem weight, and the total flavonoid content is 62.07- ± 39.76 mg QE/g dry stem weight. *T. crispa* crude extract and its isolated fraction significantly stimulate RAW264.7 cell viability (*P ≤* 0.05) and intracellular INF-γ, IL-6, and IL-8 expressions. The results of LC-MS show that four of the active compounds detected in the *T. crispa* isolated fraction are cordioside, quercetin, eicosenoic acid (paullinic acid), and boldine.

**Conclusions:**

The results of this study obviously indicate that *T. crispa* has immunomodulatory effects through the stimulation of INF-γ, IL-6, and IL-8 expressions. LC-MS phytochemical analysis showed that the *T. crispa* fraction has cordioside, quercetin, eicosenoic acid (paullinic acid), and boldine, which may be responsible for the immunostimulator effect of *T. crispa*.

## Background

The immune system is a remarkably developed defense system that protects vertebrates from foreign bodies. The immune system produces many cells and molecules that distinguish and eliminate foreign and unwanted agents. The modulation of the immune system refers to any alteration in the immune response, including stimulation, expression, amplification, or inhibition of any portion or stage of the immune response. Therefore, immunomodulators are substances used for their effects on the immune system. Immunomodulators are grouped into two types based on their effects: immunostimulators and immunosuppressors. These immunomodulators mount an immune response or defend against pathogens or tumors [1]. Immunomodulators are substances that modify the response of the immune system to a threat. Immunomodulators modulate and potentiate the immune system, keeping it highly prepared for any threat [2].

Plant extracts are commonly investigated in different parts of the world for their possible immunomodulatory properties [3]. For example, *Acorus calamus* rhizome extract inhibits the growth of several human and mouse cell lines. This extract also inhibits nitric oxide, interleukin-2 (IL-2) and tumor necrosis factor-α (TNF-α) production, as well as downregulates CD25 marker expression [4]. Many plant-derived compounds, such as sterols, sterolins, polysaccharides, alkaloids, flavonoids, lectin, and glycoproteins, are used as immunomodulators [5,6].

The modulation of the immune response to treat diseases has long been attracting attention. Numerous recent studies have made advances in the research on ethnomedicinal plants as immunomodulatory agents. Immunopharmacology is a relatively new developing branch of pharmacology that aims to identify immunomodulators. The possible uses of immunomodulators in clinical medicine include the reconstruction of immune deficit, such as AIDS treatment, and the suppression of normal immune response or exaggerated immune response in autoimmune diseases. Medicinal plants and their active components are important sources of immunomodulators. The development of immunomodulatory and anti-tumor drugs from natural compounds has attracted considerable interest [3].

Plants provide humans with food, dyes, perfumes, gum, fibers, resins, and many other useful products. Ethno-pharmacologists are currently paying more attention to the investigation of the bioactive properties and phytochemical analysis of plants for the treatment of various diseases. Several medicinal plants serve therapeutic functions in various diseases [7].

Many studies recently endorsed the use of traditional medicinal plants to treat diseases. Plants reportedly possess various biological activities [8]. Genus *Tinospora* has been known as a traditional medicine in Southeast Asia. *Tinospora* has long been used in India as medication and in the preparation of a starch known as *gilae-ka-sat* or *palo. Tinospora* is a tonic, diuretic, and antiperiodic. *Tinospora crispa*, which is abundant in the Philippines under the name *makabuhay*, which means “You may live”, can be easily used for general weakness, malarial fevers, and chronic rheumatism. The whole plant has the bitter alkaloid colombine, flavonoids, and a glucoside [9]. Previous studies were performed to evaluate the nutritional components and mineral content of *T. crispa* stems*.* Results reveal that *T. crispa* contains high moisture content of approximately 77.9% and carbohydrate content of about 19.4%. Fat, protein, fiber, and ash are present in the plant at low percentages. The most abundant elements in the plant are calcium and potassium. Other trace elements, such as silicon, magnesium, chlorine, and phosphorus, are very low. The result also shows that *T. crispa* extracts have high antioxidant property. This result is confirmed by the existence of phenolics and flavonoids, such as catechin, luteolin, morin, and rutin, in the extract. These phenolics and flavonoids are responsible for high antioxidant activity. Some studies suggest that *T. crispa* could be an important source of nutrients and natural antioxidants [10]. The various chemical components of *T. crispa* make it useful for the treatment of different diseases. Many studies support the use of *T. crispa* stem extract as an anti-parasitic. *T. crispa* stem extract has been used *in vivo* as an antimalarial agent [11]. The antioxidant properties of *T. crispa* stem extract make it useful for the treatment of diseases resulting from oxidative stress, such as cardiovascular diseases [12]. *T. crispa* is traditionally known as an anticancer drug, such that many studies investigated the anticancer activity of the plant. *T. crispa* has dose-dependent anti-proliferative activity against MDA-MB-231 cancer cells, HeLa cells, and human mammary cancer cells (MCF-7) [13]. The aqueous crude extract of *T. crispa* stems has anti-proliferative activity against the MCF-7 cell line [14]. The immunomodulatory potential of the isolated *T. crispa* fraction is investigated in this work.

## Methods

### Plant extraction

**Figure 1:**
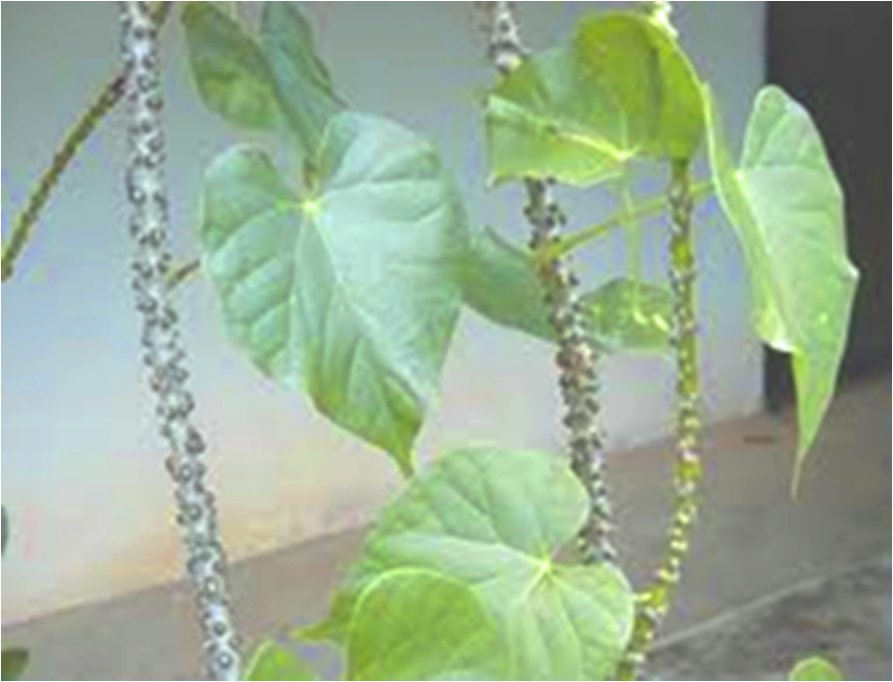
***Tinospora *** **c** ***rispa *** **stem and leaves.**

*T. crispa* dried stems; Figure 1 (harvested at the Selangor housing area in the Herbarium of University Putra in Malaysia, as voucher specimens: KLU 45568) were crushed, and 100 g of the powder was placed in a conical flask and soaked in 900 mL of 95% ethanol for 3 d at room temperature (30 ± 2°C). The suspension was occasionally shaken to completely dissolve the powder in ethanol, which was denoted by the change in colour to dark brown. The mixture was then filtered using a filter paper (Whitman, 185 mm), and distilled under reduced pressure in a rotary evaporator (Buchi, Switzerland). The extract was stored at -20°C prior to use [15].

### Profiling and fractionation of crude extracts

First, 1 g of ethanol crude extract was dissolved in 5 mL of methanol and was subjected to column chromatography fractionation in 3.0 × 50 cm glass columns (Kontes Scientific Glassware, Vineland, NJ, USA) packed with silica gel G60, 70–230 mesh (Merck, Darmstadt, Germany) linked to an EYEL-L4 pump (Tokyo Rikakikai, Tokyo, Japan). Gradient concentrations of solvents that differ in polarity (25 mL each of five different concentrations: 20%, 40%, 60%, 80%, and 100%) were used to elute the crude extract. The solvents used were hexane, ethyl acetate, methanol, acetonitrile, and water in order of increasing polarity. The yield fractions were collected in clean class tubes and gathered based on the solvent used. The solvents were evaporated from the yield fractions under reduced pressure by using a centrifuge evaporator.

### Antioxidant and free radical study

#### Ferric reducing antioxidant power (FRAP)

This method was performed by preparing the working FRAP reagent as described below:

1) The 300 mmol/L acetate (pH 3.6) buffer was prepared by mixing 3.1 g of sodium acetate trihydrate (C_2_H_3_NaO_2_:3H_2_O) with 16 mL of glacial acetic acid/L of buffer solution.

2) The TPTZ solution was prepared by dissolving 10 mmol/L TPTZ in 40 mmol/L HCl.

3) We mixed 20 mmol/L FeCl_3_ × 6H_2_O with distilled water.

4) The three reagents were mixed to a 10:1:1 ratio by adding 25 mL of the acetate buffer, 2.5 mL of the TPTZ solution, and 2.5 mL of the FeCl_3_ × 6 H_2_O solution. The working solution must always be freshly prepared.

5) Standards of known Fe(II) concentrations were prepared. (FeSO_4_:7H_2_O) was run in triplicate for calibration in several concentrations from 100 μmol/L to 1000 μmol/L.

6) FRAP reagent was used as a blank.

Approximately 150 μL of the total FRAP reagent was added to each well in a 96-well (300 μL) microplate. A 20 μL portion of the sample was then added to each well in triplicate, and the absorbance at 593 nm was measured after 15 min. The relative activities of the samples were assessed by comparing their activities with that of the Fe^+2^ equivalent [16].

#### Redical scavenging activity test (DPPH)

The test was performed according to previous studies [15]. The plant extract (1 mg) was dissolved in 1 mL of 95% ethanol to make a stock solution, and five dilutions were prepared from the stock. L-Ascorbic acid standards were prepared similar to the plant extracts. Approximately 5 μL from each dilution of plant extracts and standard was added to 195 μL of DPPH, and the mixture was loaded in a 96-well plate in triplicate for each sample. Ethanol was used as a blank. The plate was incubated at room temperature for 2 h, and the absorbance was read at 517 nm.

The antioxidant activity was calculated as the DPPH radical scavenging activity (DPPH inhibition %) according to the formula:

$$\begin{array}{ll} \mathrm{DPPH}\;\mathrm{inhibition}\%= & \left[\left(\mathrm{absorbance}\mathrm{blank}–\mathrm{absorbance}\right)\right)\\ & \left(\left(\mathrm{sample}\right)/\mathrm{absorbance}\;\mathrm{blank}\right]\times 100 \end{array}$$

The graph of the concentration of the sample versus the DPPH scavenging activity percentage was plotted to evaluate IC50 (the concentration required to inhibit 50% of the DPPH radical scavenging activity).

#### Total phenolic content

The total phenolic contents were determined as described in [17], with some modifications in the solution volumes to make them suitable for the microplate. A 10 μL portion from the extract prepared at 1 mg/mL concentration was added to 100 μL of Folin–Ciocalteu reagent (prepared beforehand at tenfold dilution with deionized water), then mixed well and set aside for 5 min at room temperature. Then, 100 μL of 10% sodium carbonate was added to the mixture, which was then incubated for 2 h at room temperature. The optical density was read at 765 nm absorbance. Gallic acid was used as the standard. The standard calibration (0 mg/mL to 200 mg/mL) curve was plotted using gallic acid. The total phenolic content is expressed as gallic acid equivalent in mg/g of dry sample or mg GAE/g sample.

#### Estimation of total flavonoid content

The aluminum chloride colorimetric method was used as described by [18] to determine flavonoid content. The method was applied by mixing 0.5 mL of plant extract (1 mg/mL), 1.5 mL of methanol, 0.1 mL of 10% aluminum chloride, 0.1 mL of 1 M potassium acetate, and 2.8 mL of distilled water. The mixture was set aside at room temperature for 30 min. The absorbance of the mixture was measured at 415 nm. A calibration curve was established to calculate total flavonoid content. The calibration curve was plotted by preparing different concentrations (0 mg/mL to 200 mg/mL) of quercetin in methanol. The total flavonoid content is expressed as quercetin equivalent in mg/g of dry sample or mg QE/g sample.

### Immunomodulatory effect *in vitro*

#### Cell line

A murine macrophage cell line RAW264.7 obtained from American Type Culture Collection (ATCC, Rockville, MD) was cultured in DMEM supplemented with 4500 g of glucose/L, 110 mg of sodium pyruvate/L, 1% penicillin–streptomycin (Sigma-Aldrich, UK), and 10% heat-inactivated fetal bovine serum (FBS) (J R Scientific, Inc., USA). The cells were cultured at 37°C in a 5% CO_2_ atmosphere incubator (NuAire, Plymouth, MN, USA). The cells were subcultured when at 80% confluence, and the cell suspension was diluted to 5 × 10^5^ cells/mL for the experiments [19]. One hour before the experiments, 1 mL of fresh medium (37°C) was placed on the six-well plates (Costar, Corning, NY). The cells were cultured for 24 h.

#### Proliferation assay

The 3-(4,5-dimethylthiazol-2-yl)-2,5-diphenyltetrazolium bromide (MTT) assay (Merck, Germany) was used to determine the effect of plant extract and isolated fractions on RAW264.7 cell proliferation. The MTT test measures the capability of cells to convert MTT to formazan. The cells were plated in 96-well tissue culture plates at a density of 5 × 10^5^ cells/mL, 5000 cells/well in complete DMEM medium and incubated in triplicate in a 96-well plate at a final volume of 100 μL for 24 h at 37°C and 5% CO_2_ conditions [20]. The cells were treated with plant crude extract, and all fractions at the final concentrations of 25, 50, 100, 200, 400, 800 and 1000 μg/mL were incubated for 24 h at 37°C and 5% CO_2_. Then, 10 μL of 5 mg/mL phosphate-buffered saline (PBS) MTT solution was added to each well. After 4 h of incubation at 37°C, the media and MTT were aspirated, and 100 μL of dimethyl sulfoxide (Fisher Scientific, UK) was added to dissolve the yellow MTT tetrazolium salt produced by metabolism to acquire purple MTT formazan salt. The amount of MTT formazan salt produced is proportional to the amount of viable cells, and the cell proliferation rate is determined by measuring the absorbance at 570 nm using a microplate reader [19,21].

#### *In vitro* stimulation for intracellular cytokines production

The capability of the isolated fraction to stimulate cytokine production in the RAW 264.7 cell was evaluated. RAW 264.7 cells (1 × 10^6^ cells/mL, 6 mL/well) were incubated in a complete DMEM medium for 24 h at 37°C in 5% CO_2_[22]. The cells were treated with 1 μg/mL of LPS (*Escherichia coli* 055: B5, Difco, Detroit, MI,USA), 1 μL/mL of brefeldin A (BD GolgiPlug™), 100 μg/mL of *T. crispa* stem crude extract, and its more effective fraction 100 μg/mL (T.c F2). The cells were incubated for 6 h [19,23]. The medium was removed before treatment with 1 μg/mL of LPS, and cells were washed with 5 mL of PBS and replenished with complete medium [24]. Cells treated with 1 μg/mL LPS alone were used as the control [22]. After incubation, the cells were washed twice with PBS and resuspended in 0.5 mL of staining buffer PBS containing 1% FBS and 0.09% (w/v) sodium azide [23].

#### Flow cytometry immunostaining of intracellular cytokines

Cells were resuspended to 10^7^ cells/mL density, and 100 μL aliquots of the cell suspension were transferred into tubes for staining. The cell suspension was pre-incubated with Mouse BD Fc Block™ purified anti-mouse CD16/CD32 mAb 2.4G2 (BD Fc Block™; Cat. No. 553141) (1 μg/10^6^ cells in 100 μL) at 4°C for 15 min to reduce nonspecific immune fluorescence staining, and the cells were fixed/permeabilized with 250 μL of fixation/permeabilization solution (BD Cytofix/Cytoperm™ Plus Fixation/Permeabilization, BD Golgi Plug™ protein transport inhibitor, Cat. No.555028) for 20 min at 4°C. The cells were washed twice with 1 mL of 1 × BD Perm/Wash™ buffer, 250 × g, for 5 min at 4°C. The fixed/permeabilized cells were resuspended in 50 μL of BD Perm/Wash™ buffer and then incubated with fluorochrome-conjugated anti-cytokine antibodies, PE Anti-Mouse IL-6 (≤0.25 μg/million cells, BD Cat. No: 554401), FITC Rat Anti-Mouse IFN-γ (≤0.5 μg/million cells, BD Cat. No: 554411), and APC Rat Anti-Mouse IL-8 (≤0.5 μg/million cells, BD Cat. No: FAB2164A) at 4°C for 30 min in the dark. The cells were washed twice with 1× BD Perm/Wash™ buffer (1 mL/wash) after incubation and then resuspended in staining buffer before flow cytometric analysis.

#### Identification of active constituents

High-resolution mass spectrometry was performed to identify and characterize the active constituents in the *T.* c*rispa* fraction 2 (T. c F2) that has proliferation and stimulation effects on RAW264.7 cells. High-resolution mass spectra were recorded on an LC-Mass (Agilent technologies 6530 Accurate-Mass QTOF LC/MC), using MeOH-Water (40:60) as the eluent. The high resolution mass spectra confirms the identity of the extracted active compounds using [M + H]^+^, [M + Na]^+^, [M + K]^+^, and [M + NH_3_]^+^[25].

#### Statistical analysis

The values are presented as mean ± standard division (S.D.). The data are statistically analyzed through one-way analysis of variance (ANOVA), univariate analysis of variance, and post hoc LSD test (comparing the treated groups with control), with *P ≤*0.05 considered as statistically significant.

The flow cytometry samples were analyzed using the two-dimensional forward and side scatter of the flow cytometer and fluorescence intensity using at least 10,000 cells collected from each sample. The cytokine analysis was performed using FACS Canto II flow cytometer and FACS Diva Version 6.1.3 software (BD Biosciences).

## Results

### Quantitative and evaluation of antioxidant contents and activity

The antioxidant activity of *T. crispa*, as evaluated by FRAP and DPPH tests, are shown in Table 1. The total phenolic and total flavonoid contents are also presented in Table 1. The results show that the *T. crispa* extract has higher antioxidant activity than standard ascorbic acid. The FRAP value of the *T. crispa* extract is 11011.11 ± 1145.42 μmol Fe^+2^/g, whereas the value for standard ascorbic acid is 7951.85 ± 330.42 μmol Fe^+2^/g. The DPPH test detected 55.79 ± 7.9% DPPH inhibition, and 22 μg/mL IC50 for *T. crispa*, and 69.03 ± 9.3% DPPH inhibition and 19 μg/mL IC50 for ascorbic acid. The results reveal that the total phenolic content of the *T. crispa* extract is 213.16 ± 1.31 mg GAE/g dry stem weight and its total flavonoids content is 62.07 ± 39.76 mg QE/g dry stem weight.

**Table 1:** **Antioxidant activity values, total phenolic and total flavonoids contents for *T. crispa *** **extract**

	**FRAP assay μmol Fe + 2/mg**	**Redical scavenging assay**		**Total phenolic contents mg GAE/g**	**Total flavonoid contents mg TE/g**
		**DPPH inhibition %**	**IC50 μg/ml**		
**Ascorbic acid**	7951.85 ± 330.42	69.03 ± 9.3	19		
***T. crispa***	11011.11 ± 1145.42	55.79 ± 7.9	22	213.16 ± 1.31	62.07 ± 39.76

Data here are expressed as mean ± standard deviation, FRAP value is expressed asFe^+2^equivalents IC50 value for the DPPH assay expressed as μg/ml. Total phenolic content is expressed as Gallic acid equivalents (GAE), while the Total flavonoid content is expressed as Trolox equivalents (TE).

### Proliferation effect on the RAW264.7 cell

**Figure 2:**
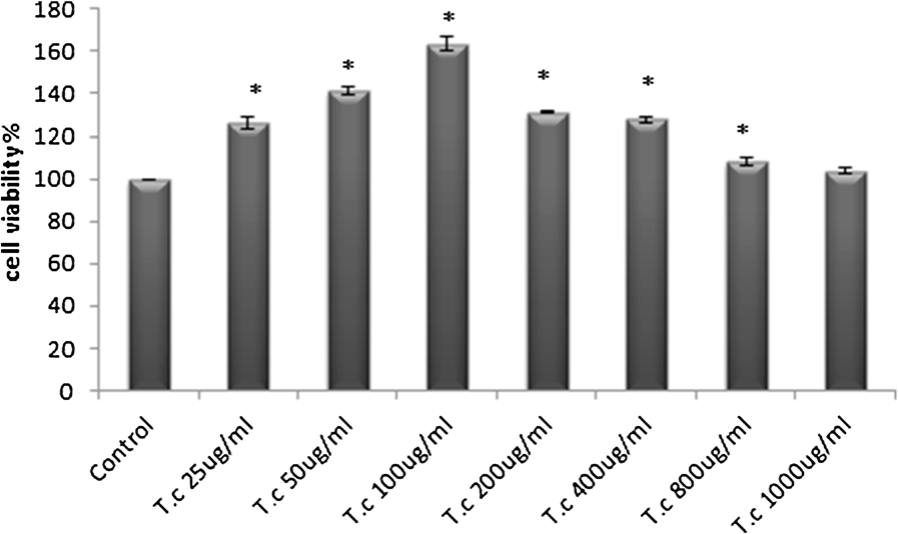
**Cell viability Percentage of RAW264.7 cell treated groups of *T. crispa *compared to control (untreated group).** Each value represents the mean percent ± S.D.*significantly different versus control group, *P ≤ 0.05*.

The immunomodulatory effect of the crude extract and five yield fractions of the plant on the RAW264.7 macrophage cell line was investigated. The results prove that *T. crispa* stimulates RAW264.7 cell proliferation in a dose-dependent manner. Cell viability significantly increased (*P ≤* 0.05) with mean viable cell percent ± SD values of 126.43 ± 2.91, 141.59 ± 1.8, 163.62 ± 3.26, 131.28 ± 0.65, 127.96 ± 1.45, and 108.18 ± 1.98 for 25, 50, 100, 200, 400, and 800 μg/mL *T. crispa* doses, respectively. The results show no significant increase in the cell viability at 1000 μg/mL *T. crispa* (103.79 ± 1.79) dose after incubation for 24 h compared with the control (Figure 2).

**Figure 3:**
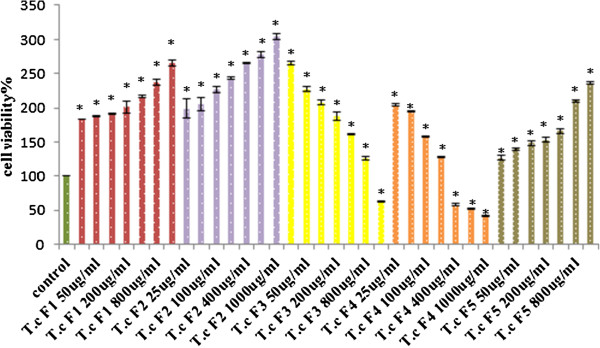
**Cell viability Percentage of RAW264.7 cell treated groups of *T. crispa *(T. c) F1, F2, F3, F4 and F5 compared to control (untreated group).** Each value represents the mean percent ± S.D.*significantly different versus control group, P ≤ 0.05.

The immunomodulatory effect of the isolated fractions on the RAW264.7 cell line was investigated, and the best fraction was chosen to investigate its effect on the intracellular expression of cytokines and was subjected to LCMS profiling to identify the active constituents. The effects of the five fractions of the plant on RAW264.7 cell line proliferation were investigated. The isolated fractions appear to have different effects on RAW264.7 cell proliferation. The *T. crispa* (F1, F2, and F5) treatments show significant dose-dependent cell viability increase *P* ≤ 0.05. The viability percentage significantly decreased *P ≤ 0.05* in a dose dependent manner compared with the control untreated cells after the treatment of 24 h incubation with *T. crispa* (F3 and F4) (Figure 3).

### *In vitro* stimulation of intracellular cytokines production

**Figure 4:**
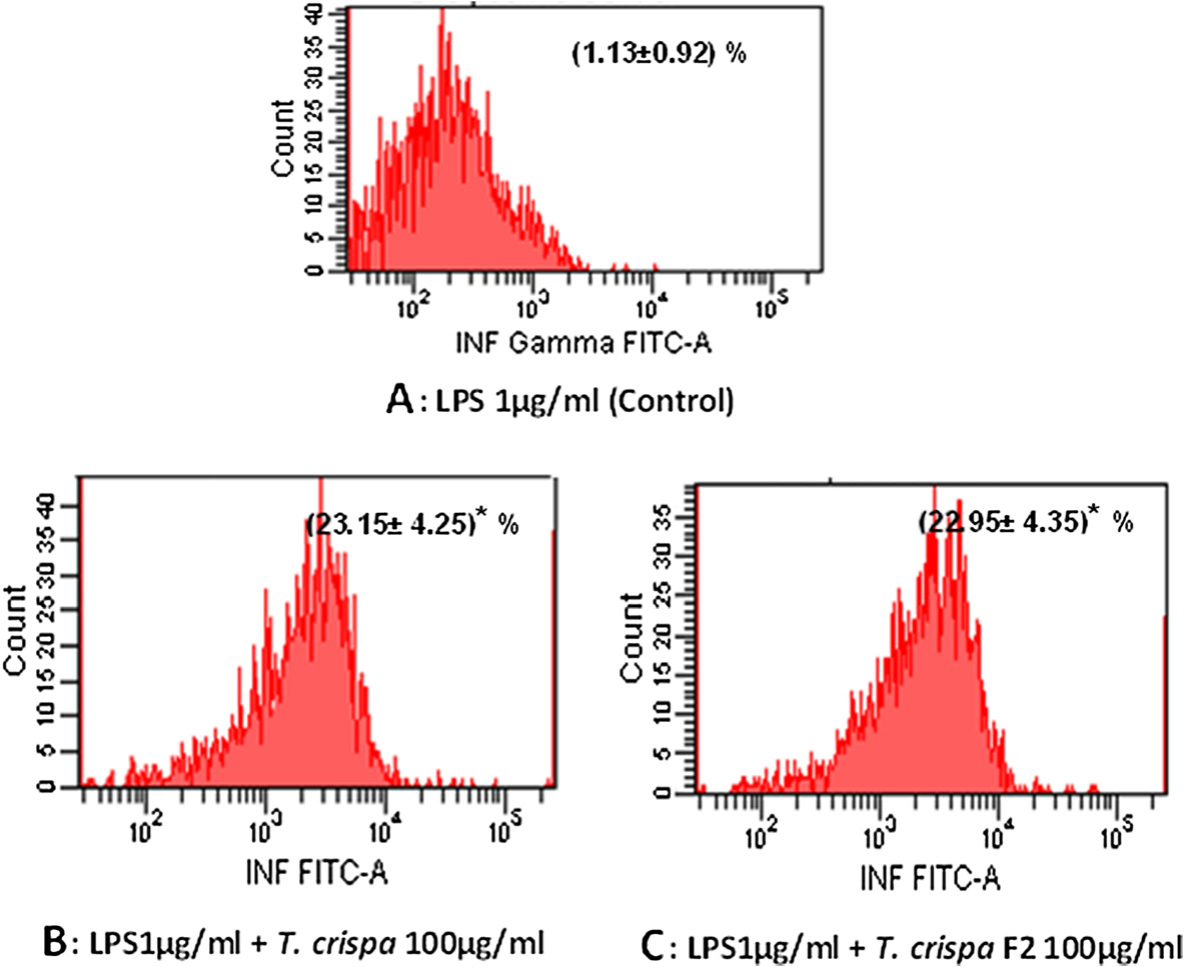
**Intracellular expression of INF-γ on RAW264.7 macrophage cell.** Flow cytometry analysis was used to assess the intracellular expression of INF-γ. The figures show the expression percent of INF-γ on RAW264.7 cell stimulated with **(A)** 1 μg/ml LPS alone as a control. **(B)** 1 μg/ml LPS + *T. crispa* 100 μg/ml. **(C)** LPS1μg/ml + *T. crispa* F2 100 μg/ml. The number was represented the mean percent of the cell ± SD. *Significant P ≤ 0.05 versus control.

The stimulation of intracellular INF-γ, IL-6, and IL-8 production were measured by flow cytometry on the RAW264.7 macrophage cell stimulated with 1 μg/mL of LPS and 100 μg/mL of each *T. crispa* extract and *T. crispa* F2. The intracellular INF-γ expression significantly (*P* ≤ 0.05) increased RAW264.7 cell stimulation with *T. crispa* extract and *T. crispa* F2 at mean percent ± SD 23.15 ± 4.25 and 22.95 ± 4.35, respectively, compared with the control RAW264.7 cells treated with LPS alone (Figure 4).

**Figure 5:**
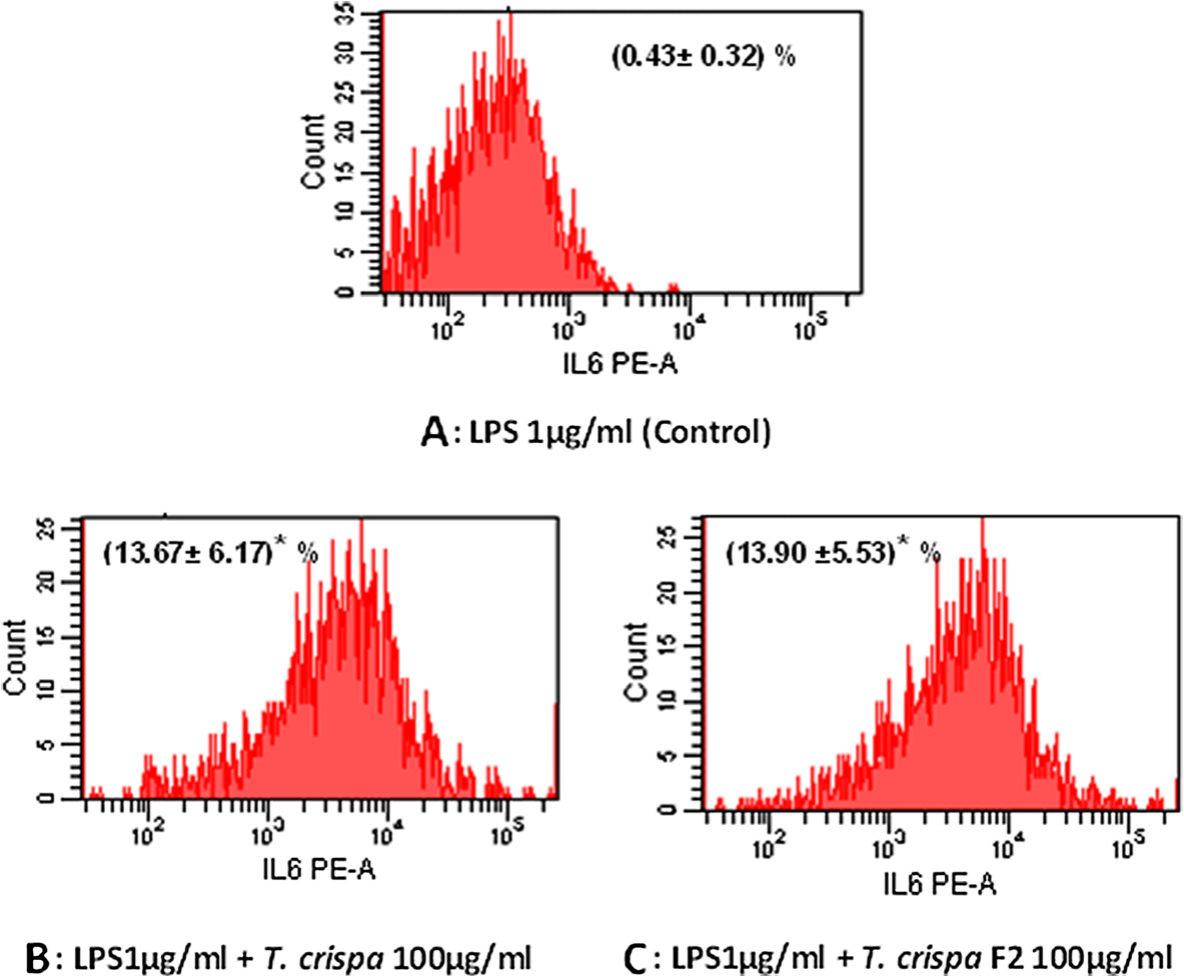
**Intracellular expression of IL-6 on RAW264.7 macrophage cell.** Flow cytometry analysis was used to assess the intracellular expression of IL-6. The figures show the expression percent of IL-6 on RAW264.7 cell stimulated with **(A)** 1 μg/ml LPS alone as a control. **(B)** 1 μg/ml LPS + *T. crispa* 100 μg/ml. **(C)** LPS1μg/ml + *T. crispa* F2 100 μg/ml. The number was represented the mean percent of the cell ± SD. *Significant P ≤ 0.05 versus control.

**Figure 6:**
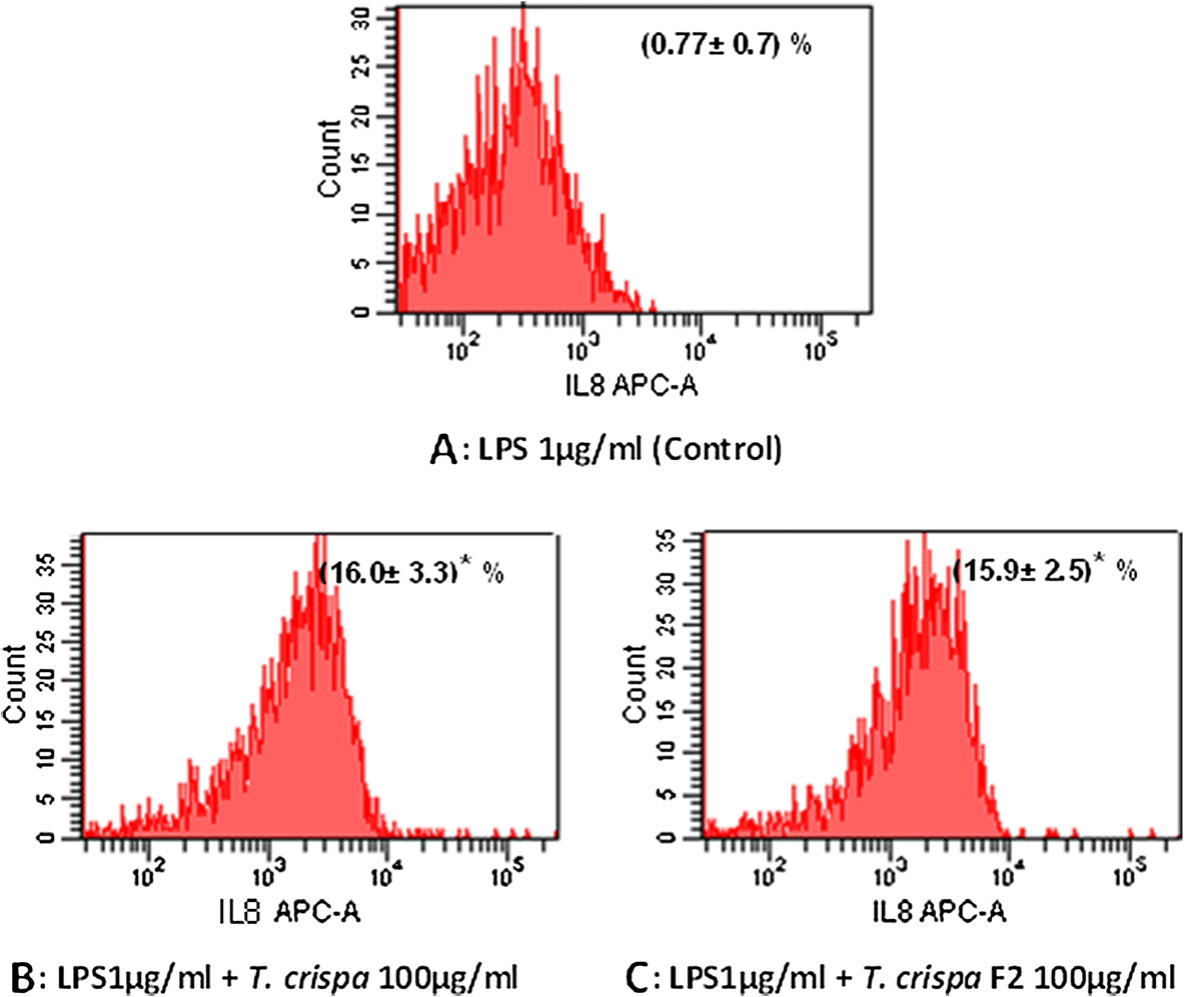
**Intracellular expression of IL-8 on RAW264.7 macrophage cell.** Flow cytometry analysis was used to assess the intracellular expression of IL-8. The figures show the expression percent of IL-8 on RAW264.7 cell stimulated with **(A)** 1 μg/ml LPS alone as a control. **(B)** 1 μg/ml LPS + *T. crispa* 100 μg/ml. **(C)** LPS1μg/ml + *T. crispa* F2 100 μg/ml. The number was represented the mean percent of the cell ± SD. *Significant P ≤ 0.05 versus control.

The results also show significant (*P ≤ 0.05*) increase in intracellular IL-6 and IL-8 expressions with the RAW264.7 cells treated with *T. crispa* extract and *T. crispa* F2. Figure 5 shows that the mean percent ± SD for intracellular IL-6 expression are 13.67 ± 6.17 and 13.90 ± 5.53 for *T. crispa* extract and *T. crispa* F2, respectively and Figure 6 shows that the mean percent ± SD for intracellular IL-8 intracellular are 16.0 ± 3.3 and 15.9 ± 2.5 for *T. crispa* extract and *T. crispa* F2, respectively, compared with the control RAW264.7 cells treated with LPS alone.

### Investigation of active constituents

**Figure 7:**
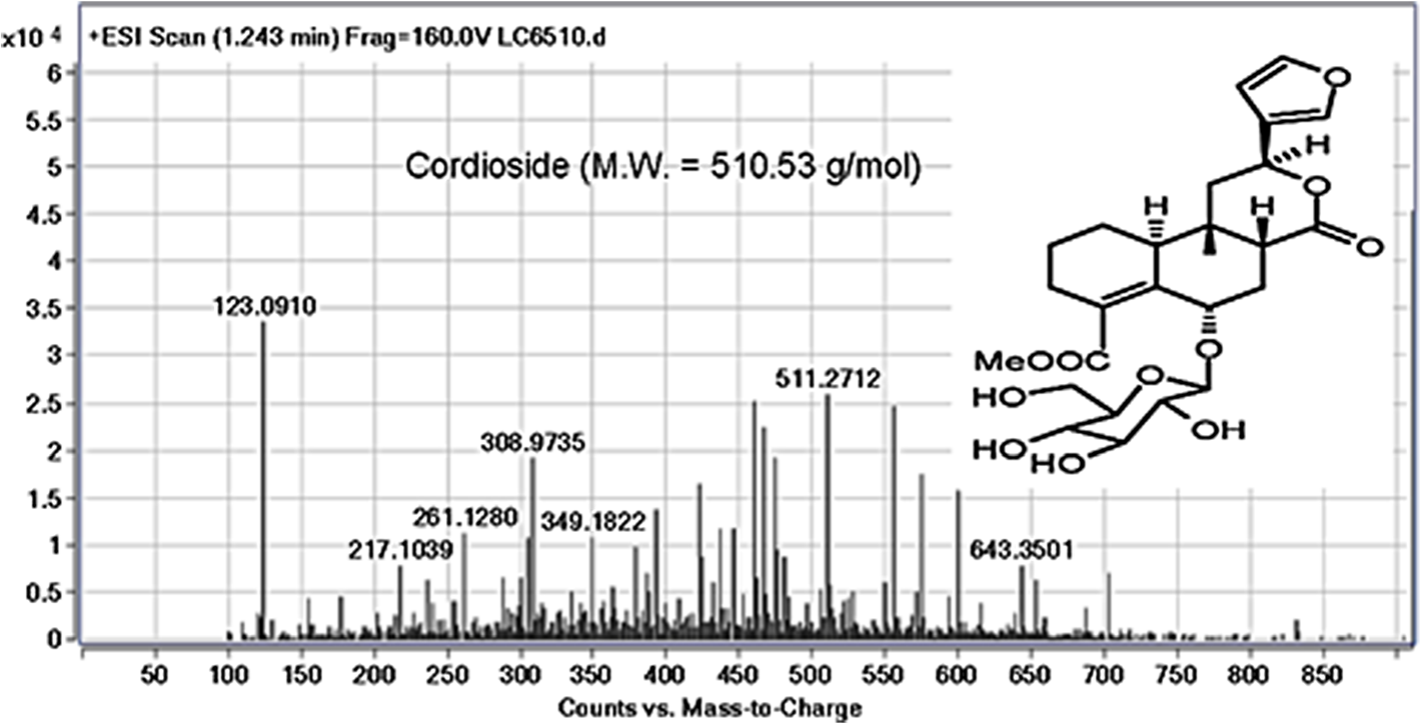
**Mass spectrum (TOF MS ES+) and chemical structure of cordioside (peak No. 1) identified in *T. crispa *** **F2.**

**Figure 8:**
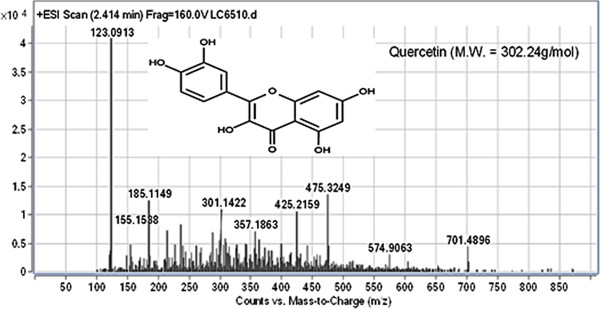
**Mass spectrum (TOF MS ES+) and chemical structure of quercetin (peak No. 2) identified in *T. crispa *** **F2.**

**Figure 9:**
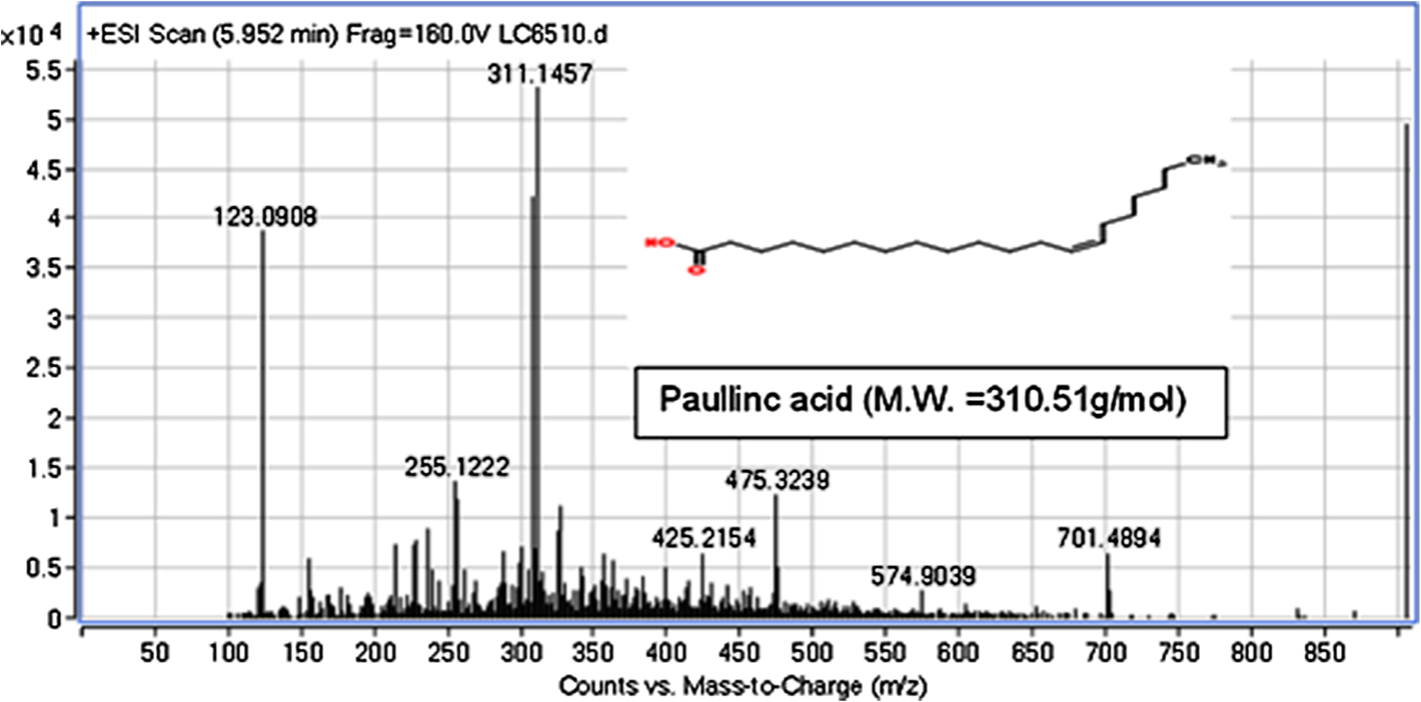
**Mass spectrum (TOF MS ES+) and chemical structure of Eicosenoic acid (paullinic acid) (peak No. 3) identified in *T. crispa *** **F2.**

**Figure 10:**
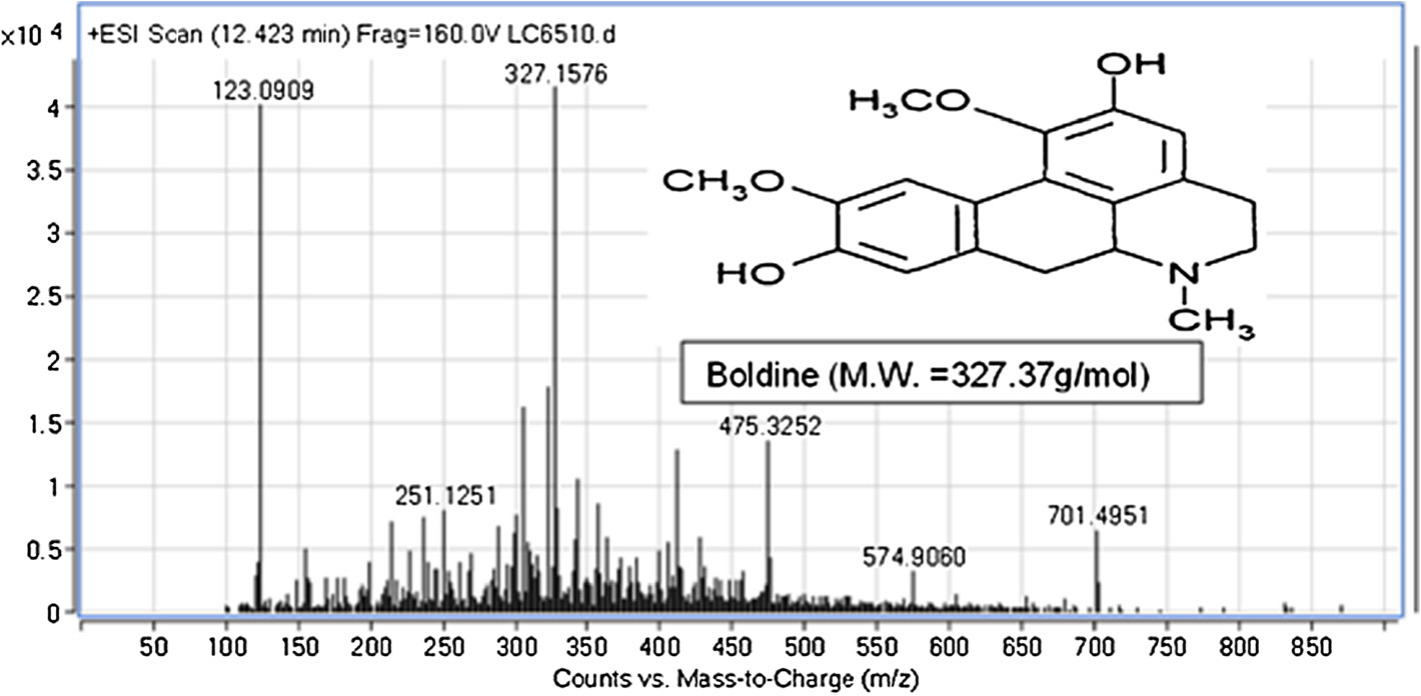
**Mass spectrum (TOF MS ES+) and chemical structure of boldine (peak No. 4) identified in *T. crispa *** **F2.**

LC-MS was performed to identify the phenolic constituents and other active compounds of *T. crispa* fraction 2. Results show that approximately four compounds were detected for *T. crispa* F2. The results show all peaks detected with their retention times, observed *m/z* and the *m/z* of fragment ions. The *T. crispa* F2 contains cordioside at *m/z* 511.2712 (Figure 7); quercetin at *m/z* 301.1422 and its fragments at *m/z* 123.0913, 185.1149, and 155.1538 (Figure 8); eicosenoic acid (paullinic acid) at *m/z* 311.1457 (Figure 9); and boldine at *m/z* 327.1576 and its fragment at *m/z* 251.1251 (Figure 10).

## Discussion

The modulation of immune response to combat diseases has long been a topic of interest. Many recent studies made progress in the research on ethnomedicinal plants as immunomodulatory agents. Immunopharmacology is a relatively new and developing branch of pharmacology that aims to discover immunomodulators. The possible uses of immunomodulators in medicine include the rehabilitation of immune deficiency. Plant extracts have been widely investigated in different parts of the world for their possible immunomodulatory properties. Some studies demonstrate the isolation of potential bioactive molecules [3].

Macrophages are the first line of defense in the innate immunity against microbial infection, and phagocytes engulf and kill microorganisms and present antigens that elicit adaptive immune responses [26]. Macrophages serve an important function in tissue remodeling through development, wound healing, and tissue homeostasis. Macrophages are essential to the innate immunity and pathology of tissue injury and inflammation [27] through phagocytosis. Macrophages secrete cytokines, such as interleukins, TNF-α, and INFs, as well as inflammatory mediators, such as nitric oxide [28]. Macrophages maintain homeostasis and serve an important function in the host defense against pathogens and attacking cells, such as cancer cells [29].

Plant extracts are widely investigated in different parts of the world for their possible immunomodulatory properties. Some studies demonstrated the isolation of potential bioactive molecules [3]. *Shosaiko-to*, a Japanese herb, possesses many ethopharmacological effects, such as the modulation of numerous host immune responses. Researchers suggest that its effect on hepatitis B might be attributed to its capability to stimulate INFs and activate natural killer cell activity [30].

The effects of *T. crispa* and its isolated fractions on inducing macrophages to release immunomodulatory cytokines, such as INF-γ, IL-6, and IL-8, were determined. RAW 267.4 macrophage cells were used in this study to determine the immunomodulatory activities by determining their intracellular cytokine production. The results of this study show that *T. crispa* and its isolated fraction modulate immunity by increasing RAW 264.7 macrophage cell proliferation in a dose-dependent manner and by significantly inducing the intracellular expression of cytokines INF-γ, IL-6, and IL-8. These findings clearly indicate the significant immunomodulatory effect of the both the plant and its active fraction as immunostimulators. These findings importantly show that the isolated fraction from *T. crispa* is a very suitable candidate for modulating macrophage function and inducing the immune system. The literature shows that immunomodulatory action is important in antitumor activity [31]. Therefore, the isolated active fraction from *T. crispa* in this study is a potential candidate for antitumor treatment. Preliminary studies on the antiproliferative efficiency of the *T. crispa* extract support this hypothesis [32].

The antioxidant activity in the plant has gained significant attention in recent years for its function in protecting cells from damage by oxidative stress. The secondary metabolites of plants are the most biologically active natural compounds. Therefore, plants are sources of food antioxidants, such as vitamin E, vitamin C, phenolic acids, carotenes, and phytoestrogens. Polyphenol or phenolic compounds are secondary metabolites composed of one aromatic ring attached to one or more hydroxyl groups. The molecules range from the simplest molecules of phenolic acid to more complex compounds (polymerized compounds), such as the flavonoids, lignins, and tannins [33,34]. Plants are the major natural sources of polyphenols (flavonoids and non- flavonoids), which give color and flavor to the plants [35]. Phenolic compounds are important in plants because the compounds contribute to the defense against microbes. Flavonoids are involved in UV filtration, symbiotic nitrogen fixation, and floral pigmentation, aside from acting as chemical messengers and regulators of physiological functions. Some flavonoids act as growth inhibitors of organisms that cause plant diseases [36]. *T. crispa* extract has high antioxidant potential, aside from the high total phenolic and total flavonoid content of the *T. crispa* extract. Many previous studies reported the correlation between the antioxidant activity of medicinal plants and their immunomodulatory potential. The phenolic and flavonoid compounds in plants have high antioxidant properties and immunomodulatory effects [37]. The exact mechanism remains unclear, but a large body of evidence supports the influence of antioxidant properties on immunomodulation. *In vitro* and *in vivo* studies demonstrated the regulatory effect of phenolic compounds on macrophage modulation and inflammatory mediator secretion from macrophages and other leucocytes [38]. Previous reports show that long-term antioxidant intake increases the antioxidant activity in the thymus, which in turn exhibits enhanced superoxide dismutase and catalase activities [39]. These activities probably stimulate a minor modification towards a mildly oxidizing environment, which favors lymphocyte maturation. The intake of this nutrition may promote the differentiation of other immune cell subsets, such as B cells, T cells, myeloid cells, natural killer cell, and dendritic cells [40].

Inflammation is the response of tissues to injury, characterized in the acute phase through increased blood flow, vascular permeability and the accumulation of fluid, leukocytes and inflammatory mediators, such as cytokines. The immune response in the chronic phase is characterized by the development of specific humoral and cellular immune responses to pathogens at the site of tissue injury. Many soluble factors participated in leukocyte recruitment in the course of the both acute and chronic inflammation processes by increasing the expression of cellular adhesion molecules and chemoattraction. Many kinds these soluble mediators regulate the activation of resident cells, such as fibroblasts, endothelial cells, tissue macrophages, and mast cells. The substances also activated the production of inflammatory cells, including monocytes, lymphocytes, neutrophils, and eosinophils [41]. The results of this study clearly reveal that the treatments using the plant and its fraction enhance immune response and stimulate the production of essential mediator cytokines, such as INF-γ, IL-6, and IL-8, which are important in the acute and chronic inflammatory response. IL-6 is important in the stimulation of acute phase protein synthesis in the liver, acts as a growth factor for mature b cells, stimulates their final maturation onto antibody-producing plasma cells, is involved in T cell activation and differentiation, and affects the induction of IL-2 receptor expression. The functions of IL-6 in the acute phase response include chronic inflammation, autoimmunity, fibrogenesis, and endothelial cell dysfunction [42]. INF-γ affects murine Kupffer cells, stimulates macrophages and is involved in the development of Th1 cells. The cellular effects of INF-γ include the up-regulation of pathogen recognition, antigen processing and presentation, inhibition of viruses, inhibition of cellular proliferation, and modifying apoptosis and immunomodulation [43]. IL-8 is a potent neutrophil chemotactic factor. Many types of cells produce large amounts of IL-8 in response to various stimuli, such as pro-inflammatory cytokines, microorganisms and their products, as well as environmental alteration, including hypoxia and hyperoxia. IL-8 is the main mediator in neutrophil-mediated acute inflammation and has a wide range of actions on the different types of cells, including endothelial cells, fibroblasts, monocytes, lymphocytes, and neutrophils. These functions suggest that IL-8 has important roles in different pathological disorders, such as chronic inflammation and cancer [44]. A previous study [45] revealed that *T. crispa* has no effect on NK cell activity. However, this study did not suggest that *T. crispa* does not affect the other immune responses. This study shows that *T. crispa* has an immunostimulatory effect on the activated macrophage mediated by enhancing expression of cytokines INF-γ, IL-6, and IL-8. These cytokines are involved in lymphocytes cell activation, which may indirectly affect the activation of NK cell type by inducing macrophages to secret the cytokines, which consequently stimulate cell-mediated immunity.

Many previous studies similarly revealed the same effect of medicinal plants, algae, or their derivatives compounds. *Astragalus membranaceus* polysaccharide improves immune response in the host individual by inducing IL-2, IL-12, and TNF-α secretion, as well as inhibits tumor progression *in vivo*[46]. *Sargassum fusiforme* has the same effect in promoting immune response and inhibits the growth of lung adenocarcinoma in mice [47].

LC-MS was performed in this study to detect and identify the possible bioactive components in the *T. crispa* fraction that may have been responsible for its immunomodulatory effect. The LC-MS results for *T. crispa* F2 identified four compounds, including cordioside (C25H34O11), quercetin (C15H10O7), eicosenoic acid (paullinic acid) (C20H38O2), and boldine (C19H21NO4). Cordioside has been isolated from *T. crispa*, with three other compounds, tinosporaside, columbin, and β-hydroxyecdysone [48]. Many previous studies reported the isolation and identification of the phenolic compounds, alkaloids, flavonoid, diterpenes, and triterpenes from *T. crispa* and showed their biological activities as highly antioxidant compounds and antiproliferative and anti-inflammatory activities [49]. An alkaloid isolated from *T. crispa* has cytotoxic activity against *Toxoplasma gondii*[50]. Borapetoside C, isolated from *T. crispa*, improved insulin sensitivity in diabetic mice [51]. Praman et al. isolated five compounds (uridine, adenosine, higenamine, salsolinol, and tyramine) from *T. crispa* and proved the effects of these compounds on the cardiovascular system, particularly on the reduction of blood pressure [52].

Cordioside and quercetin are flavonoids that have powerful antioxidant activity and many pharmacological applications as immunostimulators and anticancer agents [53].

Eicosenoic acid (paullinic acid) is an omega-7 fatty acid present in various plants. Omega fatty acids are fatty acids that cannot be produced by the human body and are essential for human health. Thus, omega fatty acids must be acquired from food. Omega fatty acids are important in brain function, growth, and development. Research shows the effects of such acids on reducing inflammation and lowering the risk of chronic diseases, such as cancer, heart disease, and arthritis. Omega fatty acid deficiency is accompanied by symptoms, such as poor memory, fatigue, heart problems, dry skin, poor circulation, and depression [54].

Boldine is an alkaloid. Research conducted during the early 1990s showed that boldine is one of the most potent natural antioxidants. Many studies focused on the pharmacological properties of boldine. The antioxidant activity of boldine inspired researchers to investigate its effect as an anticancer, cytoprotective, anti-inflammatory, anti-atherogenic, anti-diabetic, and vasorelaxing immunomodulator [55].

## Conclusions

*T. crispa* stimulates immune activity by increasing the intracellular expression of cytokines INF-γ, IL-6, and IL-8. The *T. crispa* fraction was determined through LC-MS phytochemical analysis to contain the compounds cordioside, quercetin, eicosenoic acid (paullinic acid), and boldine, which may be responsible for the immunostimulator potential of *T. crispa*. Therefore, this plant is a candidate drug that deserves further development and pharmacological studies as a new drug.

## Competing interests

The authors declare there is no competing interest.

## Authors’ contributions

WAN is conceived, designed and performed experiments. IF participated in the Flow cytometry part of the study. WNA, MAA and SI wrote and revised the manuscript. All authors read and approved the final manuscript.

## Pre-publication history

The pre-publication history for this paper can be accessed here:

http://www.biomedcentral.com/1472-6882/14/205/prepub
